# Hydro-dispersion of subincisional cortex

**DOI:** 10.1186/s12886-022-02314-0

**Published:** 2022-02-17

**Authors:** Teruyuki Miyoshi, Hironori Yoshida, Takahiro Shimowake, Tetsuro Oshika

**Affiliations:** 1 Miyoshi Eye Clinic, Hiroshima, Japan; 2 Shimowake Eye Clinic, Ehime, Japan; 3 Department of Ophthalmology, Faculty of Medicine, University of Tsukuba, 1-1-1 Tennoudai, Tsukuba, Ibaraki 305-8575 Japan

**Keywords:** Cataract surgery, Cortex, Subincisional, Irrigation and aspiration

## Abstract

**Background:**

A simple technique to facilitate removal of subincisional cortex in cataract surgery is presented.

**Methods:**

A disposable 27-gauge blunt needle attached to a 5.0-ml syringe containing balanced salt solution (BSS) is introduced through the side port incision into the anterior chamber. The tip of the needle is directed toward the capsule fornix beneath the incision site, and BSS is flushed to disperse the remaining cortex. Thereafter, the coaxial irrigation/aspiration device is used to remove the loosened cortex.

**Results:**

This technique was used in 60 eyes of 60 patients with difficulty of removing cortical remnant in the subincisional space. Subincisional cortical material was successfully removed in 93.3% (56/60 eyes). There were no intraoperative and postoperative complications related to this procedure.

**Conclusions:**

The hydro-dispersion technique is a simple and safe approach to remove the subincisional cortical material that is difficult to manage with the standard coaxial irrigation/aspiration device.

**Supplementary Information:**

The online version contains supplementary material available at 10.1186/s12886-022-02314-0.

## Introduction

Removal of cortical material beneath the incision site remains challenging even in modern cataract surgery. Bimanual irrigation and aspiration techniques have been developed for safer removal of subincisional cortex [1–5]. This method, however, entails creation of additional side port wound and special irrigation/aspiration cannulas are required. In addition, intraoperative conversion from a coaxial irrigation/aspiration handpiece to bimanual irrigation/aspiration cannulas add one extra step to the surgical procedures. The hybrid bimanual technique [6], that uses the coaxial handpiece for irrigation and bimanual aspiration handpiece for aspiration, has similar disadvantages.

In 2002, Dewey reported a technique to use J-cannula to hydrodissect the cortex from the posterior capsule in routine cataract surgery [7]. He introduced the cannula through the main incision and irrigated the cortex from the center to periphery before performing irrigation/aspiration of the cortex. In 2018, Lake et al. described a technique called second-wave hydrodissection in femtosecond laser-assisted cataract surgery [8]. They performed second hydrodissection from the center toward opposite the point of entry of the cannula after phacoemulsification and before irrigation/aspiration of the cortex. These methods were developed to separate the intact cortical bowl from the capsular bag and were not designed to remove subincisional cortex which was left after irrigation/aspiration of cortical bowl.

We herein present a different method, the hydro-dispersion technique, to flush and loosen the subincisional cortex and report the results of clinical evaluation of its efficacy in cases with difficulty of removing cortical remnant in the subincisional space.

## Methods

**Fig. 1:**
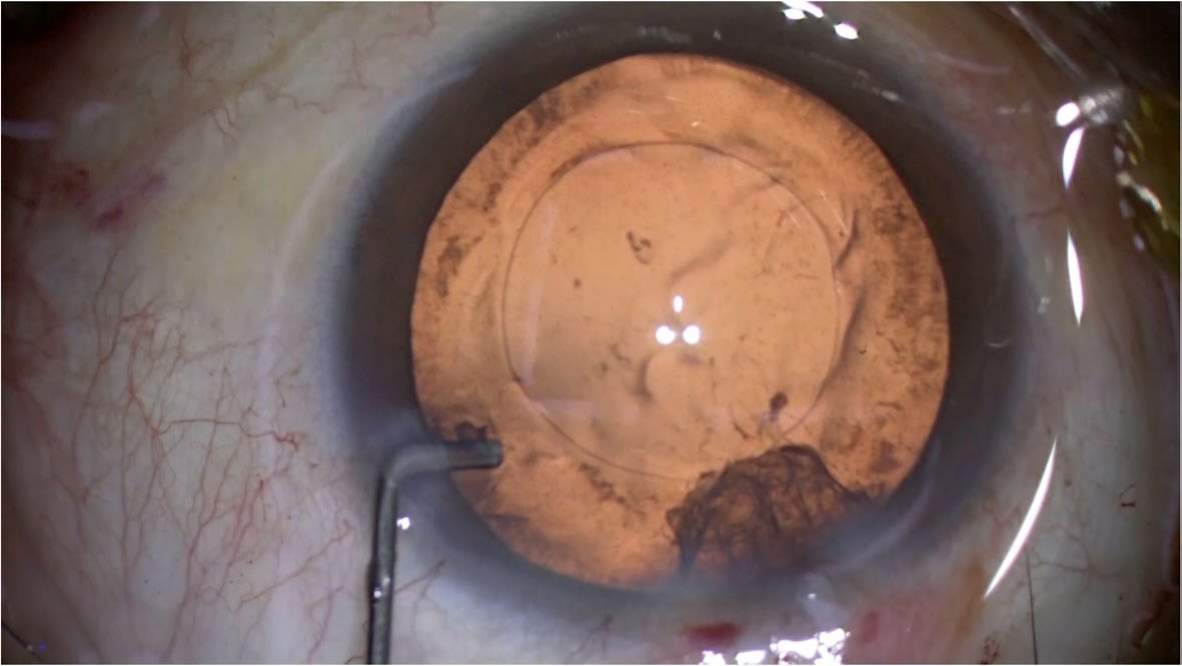
A disposable 27-gauge blunt cannula attached to a 5.0-ml syringe containing balanced salt solution is introduced through the side port incision into the anterior chamber

**Fig. 2:**
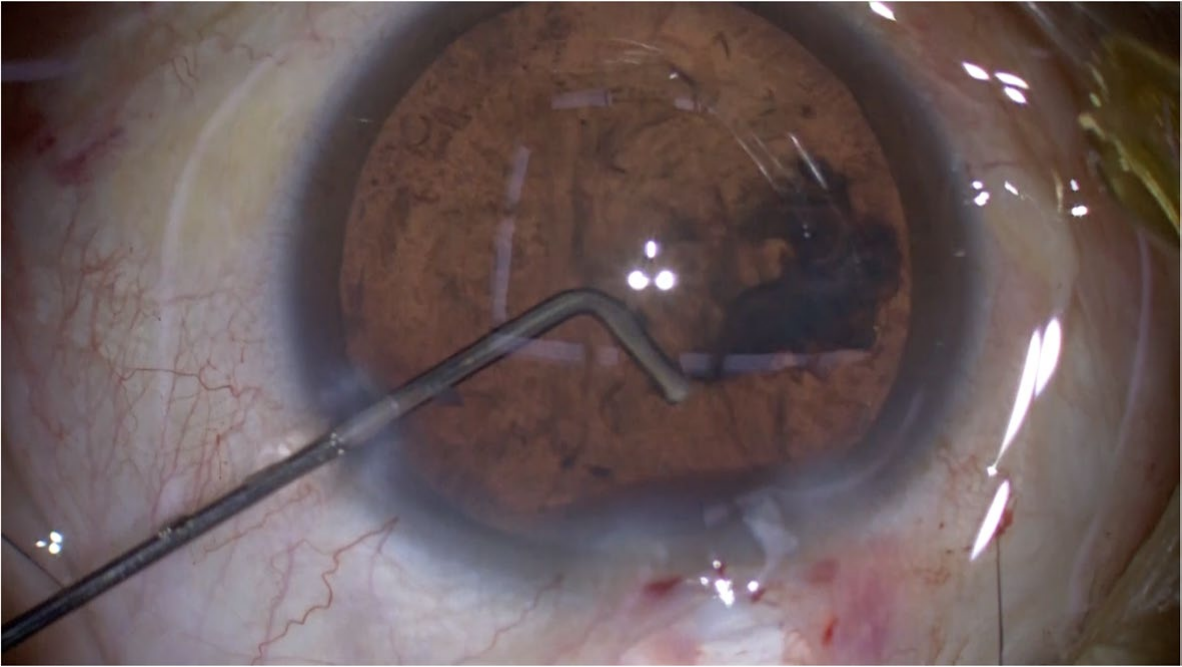
The tip of the needle is directed toward the subincisional site, and balanced salt solution is flushed to disperse the remaining cortex

**Fig. 3:**
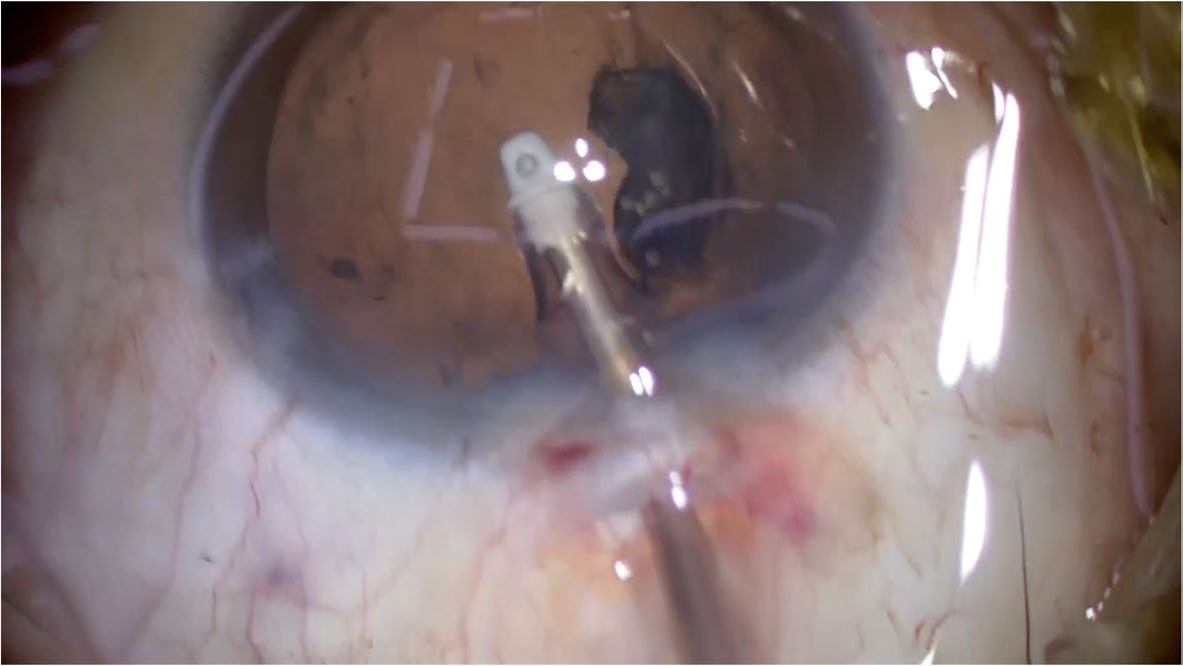
The coaxial irrigation/aspiration device is used to remove the loosened cortex

When removal of subincisional cortex was difficult with a coaxial irrigation/aspiration handpiece during routine phacoemulsification and intraocular lens (IOL) implantation, this technique was applied. The 2 ~ 3 mm tip of a disposable 27-gauge straight blunt needle (Nipro disposable ophthalmic needle 00–222, Inami) is bended about 90 degrees with a needle forceps. The needle attached to a 5.0-ml syringe containing balanced salt solution (BSS) is introduced through the side port incision into the anterior chamber (Fig. 1). The tip of the needle is directed toward the capsule fornix beneath the incision site, and BSS is flushed to disperse the remaining cortex (Fig. 2). The clump of cortex is dislodged from the equator and sometimes swirl as a vortex in the posterior chamber. At this point, cares should be taken not to overpressure the anterior chamber. If there is no leakage of BSS from the side port incision, the intraocular pressure may rise sharply, causing discomfort or pain of the patients. After the dispersion of cortical material, the coaxial irrigation/aspiration device is used to remove the loosened cortex (Fig. 3). Surgery was conducted at the Department of Ophthalmology, University of Tsukuba (TO) and Miyoshi Eye Clinic (TM).

A written informed consent regarding surgery was obtained from each patient before surgery. The study adhered to the tenets of the Declaration of Helsinki. The study protocol was approved by the Institutional Review Board of Miyoshi Eye Clinic (CS-032) and University of Tsukuba (R01-288). We received no outside funding to conduct the current study.

## Results

The hydro-dispersion technique was applied in a total of 60 eyes (60 patients) from September 2019 to April 2020. The patients’ demographics are shown in Table 1. The subincisional cortex was successfully removed with this technique in 56 eyes (93.3%). In four eyes (6.7%), small amount of cortex remained in the fornix of the capsular bag, which was removed using bimanual irrigation/aspiration cannulas. There were no intraoperative complications related to the performance of this technique, such as posterior capsule rupture, damage to the iris, tear at the edge of continuous curvilinear capsulorhexis, and intraocular bleeding.

**Table 1:** Demographics of patients

Male/female	24/36
Age (years)	72.5 ± 4.5 (57 ~ 83)
DCVA (logMAR)	0.314 ± 0.311
Intraocular pressure (mmHg)	15.1 ± 2.8
Corneal power (diopter)	44.39 ± 1.57
Axial length (mm)	23.62 ± 2.12
LOCS III grading	
nuclear color (1 ~ 6)	1/16/26/10/6/1
nuclear opalescence (1 ~ 6)	1/14/29/9/5/2
cortical cataract (1 ~ 5)	18/13/11/16/2
posterior subcapsular cataract (1 ~ 5)	28/13/11/6/2

Mean ± standard deviation (range), *DCVA* distance-corrected visual acuity, *logMAR* logarithm of minimum angle of resolution

Postoperatively, no eyes showed severe inflammation in the anterior chamber. There was no leakage of the aqueous humor from the main and side port incisions. At 3 months after surgery, mean distance-corrected visual acuity was -0.06 ± 0.10 (mean ± standard deviation) logarithm of minimum angle of resolution, and mean rate of corneal endothelial cell loss was 5.3 ± 1.8%. There was no postoperative complication, such as cystoid macular edema, endophthalmitis, toxic anterior segment syndrome, corneal decompensation, and malposition of an intraocular lens.

## Discussion

The hydro-dispersion technique does not require any special instruments. A disposable 27-gauge blunt needle and 5.0-ml syringe suffice to perform this procedure. It is a very safe method since only flush of BSS is applied to the equator zone of the capsule without any aspirating or polishing maneuvers involved. In 60 consecutive cases that received this technique, there were no intraoperative complications related to the performance of this technique. The wound integrity was not jeopardized and no leakage of the aqueous humor from the main or second incisions was observed. Endophthalmitis and toxic anterior segment syndrome were not seen in any cases. Retinal problems such as cystoid macular edema did not occur. Postoperative distance-corrected visual acuity was excellent, and the rate of corneal endothelial cell loss was low at 3 months postoperatively.

**Fig. 4:**
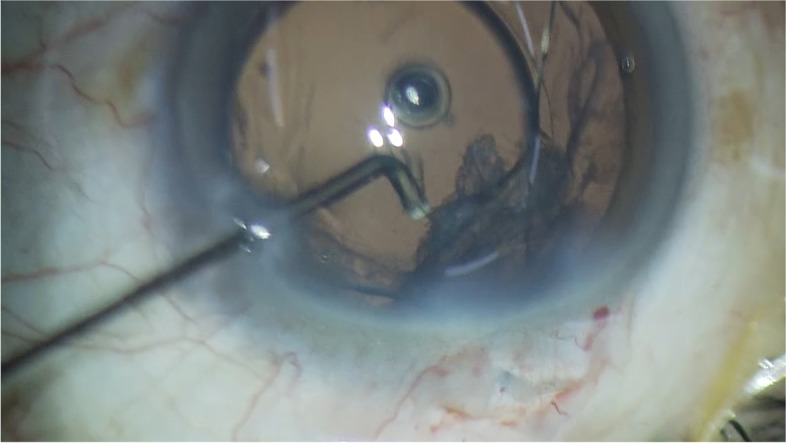
The hydro-dispersion technique can be applied after implantation of an intraocular lens

This technique can be applied in the presence of IOL. After implantation of IOL in the capsular bag, the hydro-dispersion procedure can be done to dislodge the remaining cortex (Fig. 4). The instrumentation and technique are same as described above. Another indication is the removal of cortex in the presence of a capsular tension ring. One challenge in using a capsular tension ring is the difficulty of removing cortical material once the ring is implanted [9]. The circular ring compresses residual cortical material against the capsular bag and impedes its removal. By applying the hydro-dispersion technique, the cortical material compressed by the ring is loosened, leading to safer and easier removal with the irrigation/aspiration device.

The limitation of this study is that it is not clear how much amount of cortex remnant can be dealt with this technique. In case of a large amount of residual cortex, other techniques such as bimanual irrigation/aspiration may be easier and more efficient.

The current hydro-dispersion technique affords a simple and safe approach to remove cortical material that is difficult to manage with the standard coaxial irrigation/aspiration device.

## Supplementary Information

**Additional file 1.**

## Data Availability

The datasets generated during and/or analyzed during the current study are available from the corresponding author on reasonable request.
